# Supplementary material for: Combinatorial Microgels for 3D ECM Screening and Heterogeneous Microenvironmental Culture of Primary Human Hepatic Stellate Cells
Source: Adv Sci (Weinh). 2024 Feb 13;11(15):2303128. doi: 10.1002/advs.202303128 (PMC11022709; doi:10.1002/advs.202303128)
Supplement: Supplementary file 1 — Supporting Information [file ADVS-11-2303128-s001.pdf]

ADVANCED SCIENCE
Open Access

Supporting Information

for *Adv. Sci.*, DOI 10.1002/advs.202303128

Combinatorial Microgels for 3D ECM Screening and Heterogeneous Microenvironmental Culture of Primary Human Hepatic Stellate Cells

*Hyeon Ryoo, Regina Giovanni, Hannah Kimmel, Ishita Jain and Gregory H. Underhill**

Combinatorial Microgels for 3D ECM Screening and Heterogeneous Microenvironmental Culture of Primary Human Hepatic Stellate Cells

Hyeon Ryoo, Regina Giovanni, Hannah Kimmel, Ishita Jain, Gregory H. Underhill*

Supplementary Information

Supplementary Methods

Supplementary Figure 1. Protein distribution in microgels depending on protein integration step.

Supplementary Figure 2. Porosity of microgel scaffolds in 3D space.

Supplementary Figure 3. Immunofluorescence images and quantification for lysyl oxidase and α smooth muscle actin in HSCs cultured with microgels.

Supplementary Figure 4. RT-qPCR GAPDH mRNA expression for HSCs in 3D culture with distinct microgel stiffness-ECM conditions.

Supplementary Figure 5. RT-qPCR results for HSCs cultured in microgel scaffolds for ACTA2, CDH2, IL6, LOX, PDGFRB, and TIMP1.

Supplementary Figure 6. Immunofluorescence and RT-qPCR data with NP conditions included

Supplementary Figure 7. MMP2 and Resazurin readouts with 95% confidence intervals ranked based on average value.

Supplementary Figure 8. Linear regression coefficients for MMP2 and resazurin readouts of subset data for 2 and 3 component conditions.

Supplementary Methods

Microgel Scaffold Porosity Quantification

5 µL of 4NP and 8NP microgels were diluted to a total of 25 µL of solution by addition of 20 µL of 0.1 mg/mL of 2 MDa Dextran-FITC (FD2000S-100MG, Millipore Sigma). The 25 µL solution was added onto PEG microwells and centrifuged at 300 xG for 1 min. The microwells were supplemented with 225 µL of 0.1 mg/mL 2MDa Dextran-FITC for a total of 250 µL. Microgel scaffolds were imaged using a Zeiss LSM 880 confocal laser scanning microscope with a 40x NA1.2 immersion objective and 1.33 RI oil at 25 °C. 50 slice Z-stack images with a scale of 0.21 x 0.21 x 1.00 µm voxels were taken at a resolution of 1024 x 1024 per z slice. Images were analyzed using Imaris 10.0.1 to obtain the volume occupied by the dextran-FITC. Porosity was measured as the volume with signal of FITC divided by the total volume of the 3D image.

Supplementary Figures

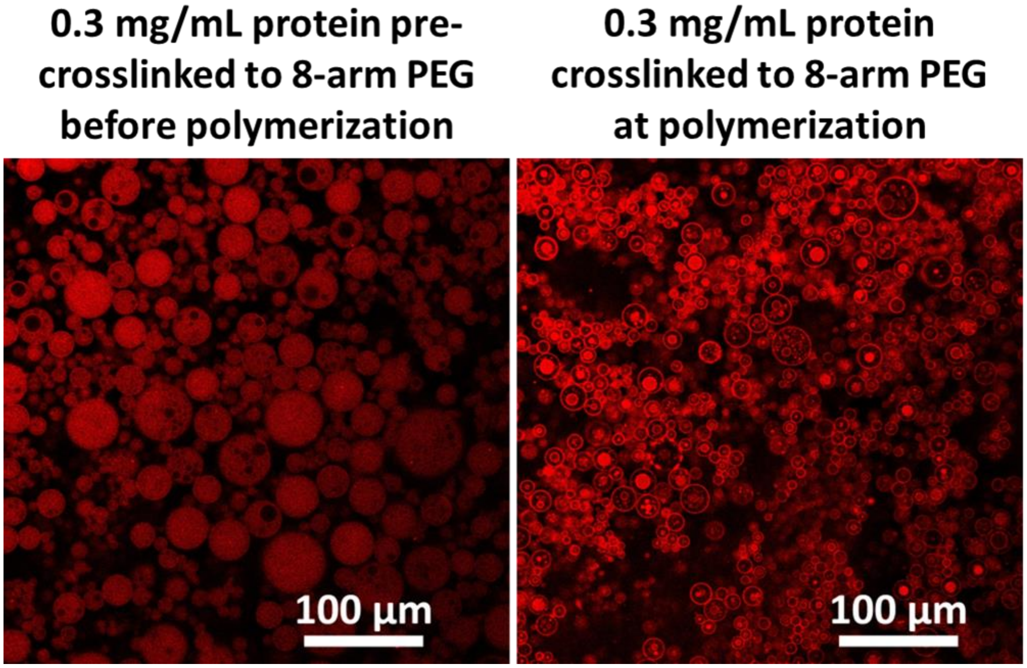

Supplementary Figure 1. Distribution of SVA-PEG-SH functionalized Alexa Fluor 555 tagged antibody in 8-arm 8% PEG norbornene microgels at 0.3 mg mL^{-1} protein and 100 µM SVA-PEG-SH final concentration. When the antibody was pre-crosslinked to the PEG norbornene, the antibody was more evenly distributed across the microgel. When the antibody was crosslinked to the PEG norbornene at the time of polymerization, the antibody tended to aggregate on the outside or core of the microgel. Red: Alexa Fluor 555 antibody.

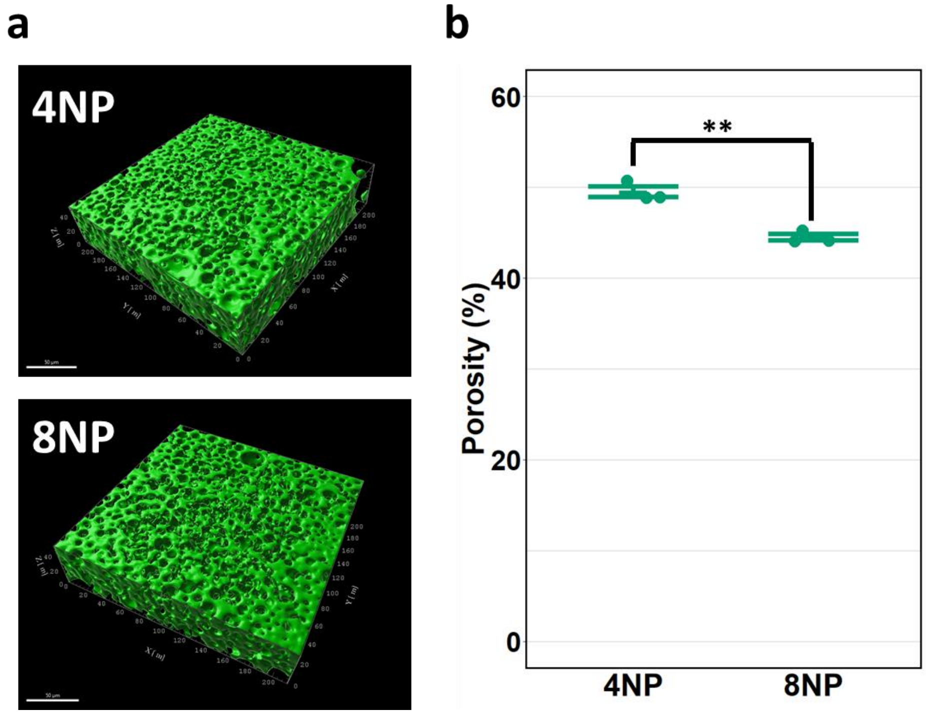

Supplementary Figure 2. **a)** Representative Imaris volumetric projection of dextran-FITC signal in imaged confocal images. Dextran-FITC signal indicates pores within the microgel scaffold. Scale bar = 50 µm **b)** Average ± standard error of means porosity of the microgel scaffolds built with 4NP and 8NP microgels. ** $p < 0.01$. Student's t-test. n = 3 experimental replicates.

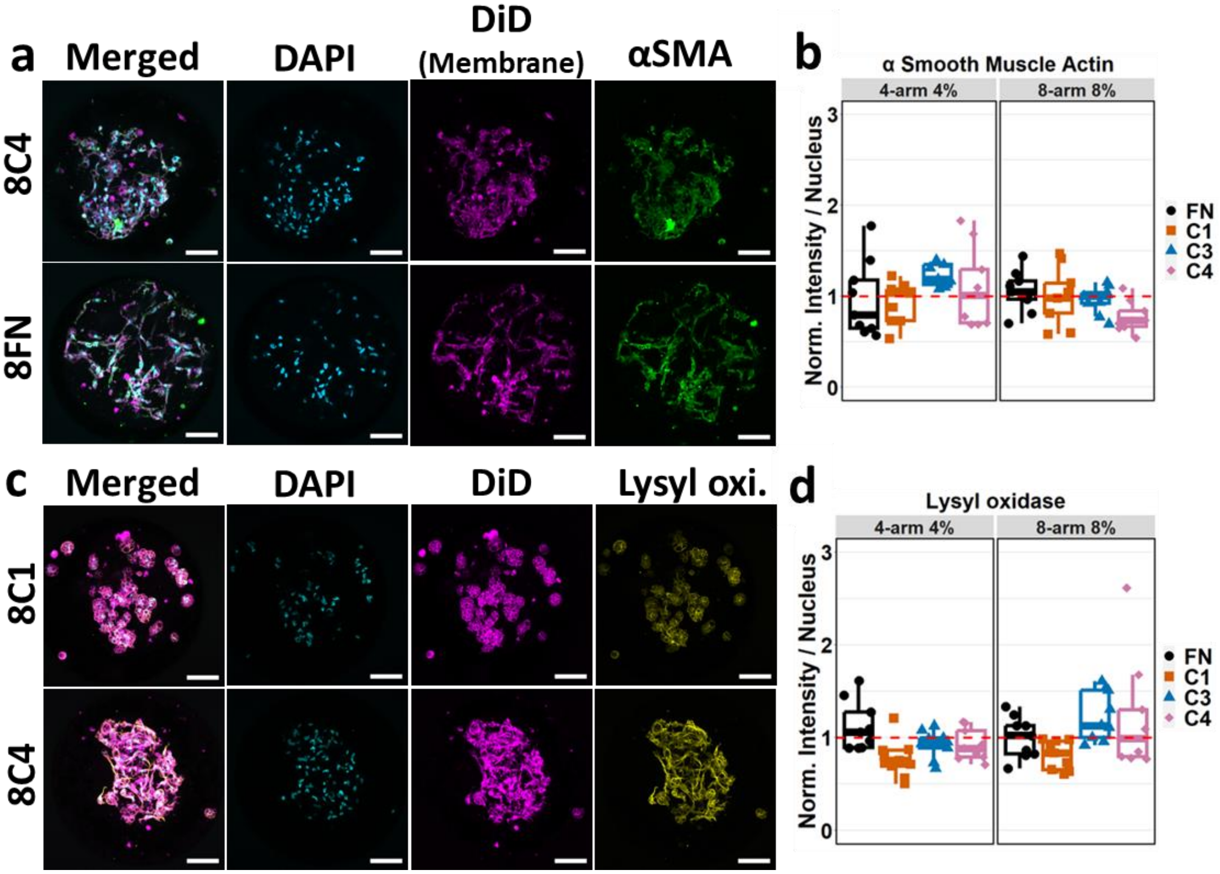

Supplementary Figure 3. a) Representative maximum intensity projection of confocal images of HSCs cultured with 8C4 and 8FN scaffold. Light blue: DAPI. Purple: DiD membrane stain. Green: anti-alpha smooth muscle actin. Scale bar: 100 µm. **b)** Box and whisker plots of the anti-alpha smooth muscle actin fluorescence intensity per nucleus normalized to the average of all conditions per experimental replicate. n = 9 from 3 experimental replicates. **c)** Representative maximum intensity projection of confocal images of HSCs cultured with 8C1 and 8C4 scaffolds. Light blue: DAPI. Purple: DiD membrane stain. Yellow: anti-lysyl oxidase. Scale bar: 100 µm. **d)** Box and whisker plots of the anti-lysyl oxidase fluorescence intensity per nucleus normalized to the average of all conditions per experimental replicate. n = 9 from 3 experimental replicates. Two-way interaction ANOVA.

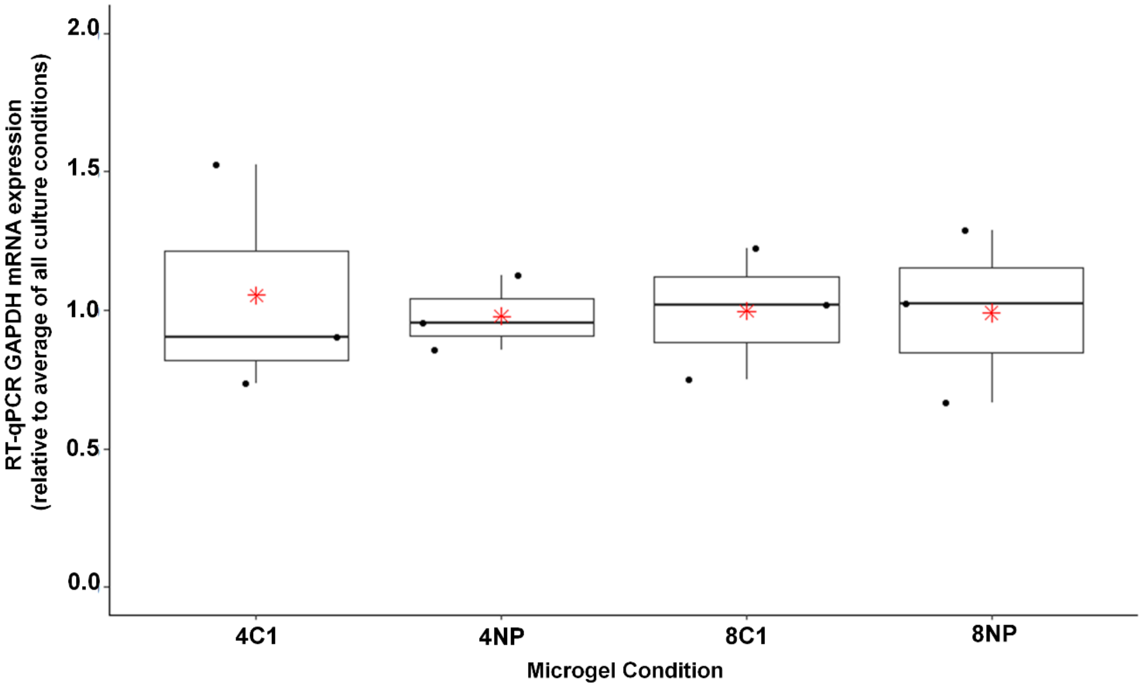

Supplementary Figure 4. RT-qPCR GAPDH mRNA expression for HSCs in 3D culture with distinct microgel stiffness-ECM conditions. mRNA expression of GAPDH was evaluated for HSCs following culture with 4 distinct microgel conditions utilized for the reported gene expression studies: (i) 4C1, (ii) 4NP, (iii) 8C1, and (iv) 8NP. GAPDH expression is stable across conditions examined. Box and whisker plots of GAPDH mRNA expression, relative to the average of all conditions. Red asterisk = mean expression for each condition. n=3 RT-qPCR experiments, including the average of 3 separate HSC/microgel co-cultures for each microgel condition. A linear model characterizing the impact of microgel culture condition on the normalized expression of GAPDH (to the bulk plate average) was created. ANOVA analysis of this linear model showed that the microgel culture condition did not significantly impact the normalized expression of GAPDH ($p = 0.9892$). Further, least square means analysis confirmed the stability of GAPDH expression across the conditions, specifically, that there were no significant pairwise differences in expression between the conditions (4C1-4NP $p = 0.9885$, 4C1-8C1 $p = 0.9951$, 4C1-8NP $p = 0.9934$, 4NP-8C1 $p = 0.9998$, 4NP-8NP $p = 0.9999$, 8C1-8NP $p = 1.0000$).

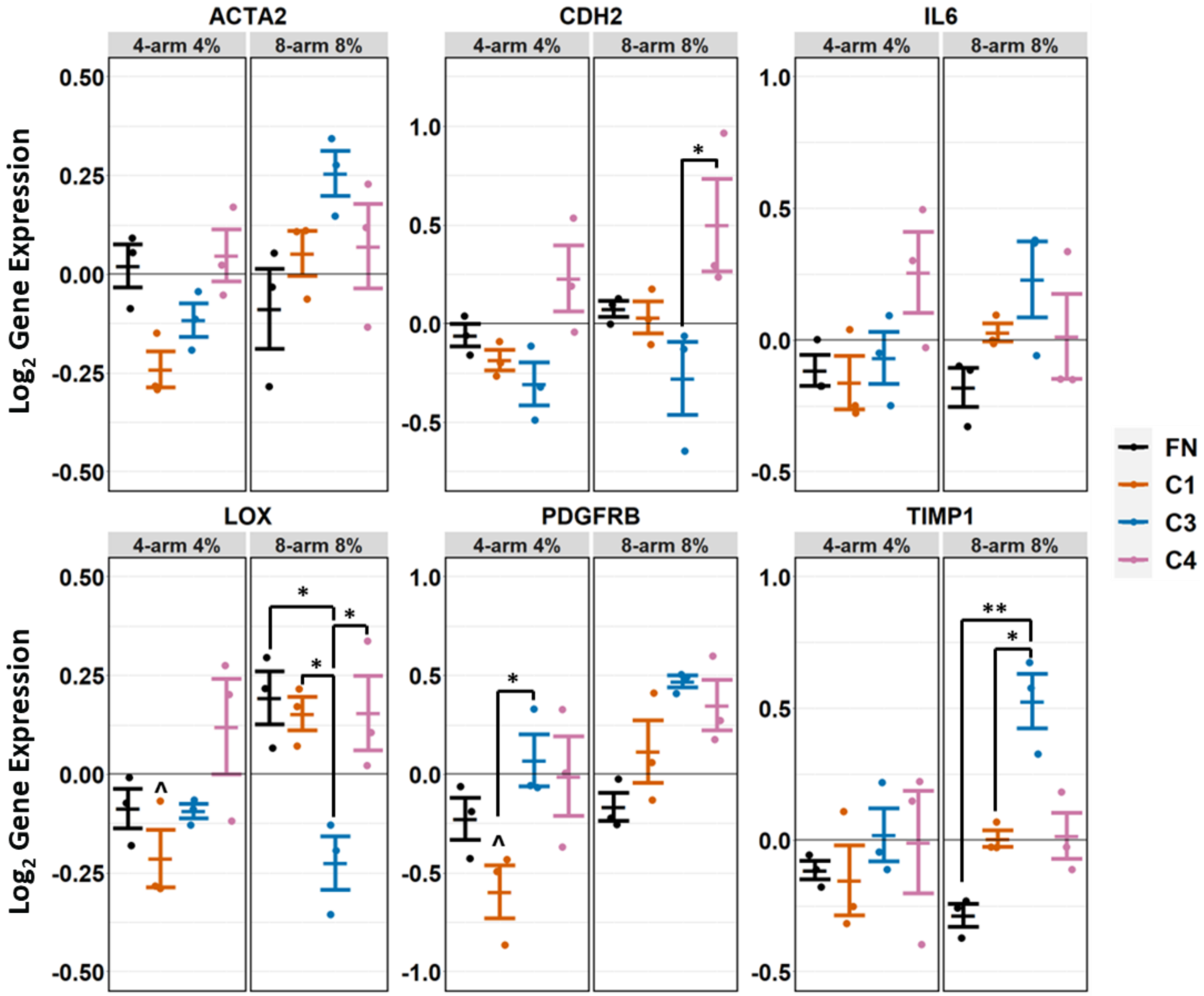

Supplementary Figure 5. Average ± standard error of means for the genes not shown individually in Figure 4. n = 3 experimental replicates. Two-way interaction ANOVA analysis. * $p < 0.05$ ** $p < 0.01$ where * means between conditions and ^ against its 8-arm 8% counterpart.

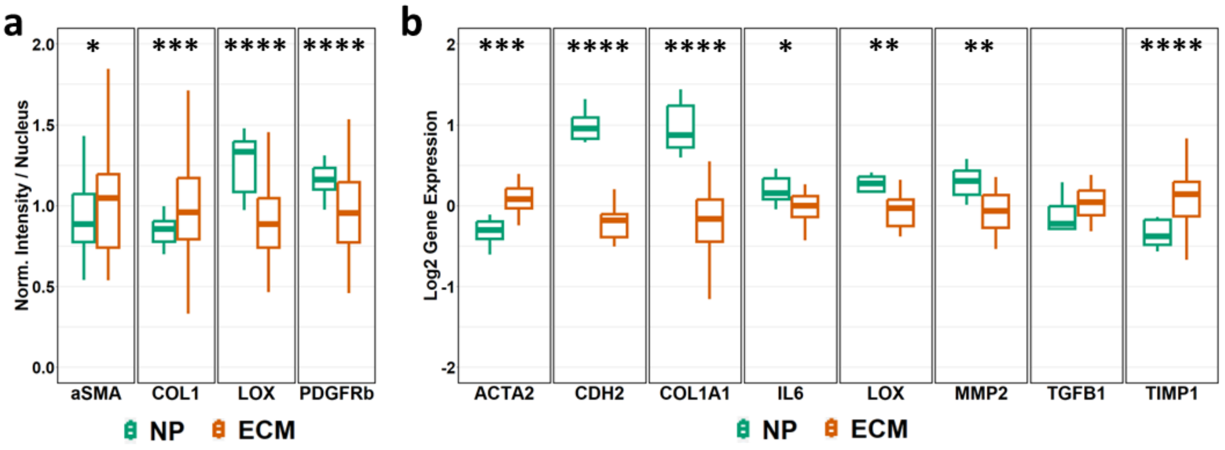

Supplementary Figure 6. Immunofluorescence **(a)** and RT-qPCR **(b)** data as shown before with the addition of no protein (NP) microgels. Values were renormalized against the average of all 10 conditions within an experiment. Student's t-test. * $p < 0.05$ ** $p < 0.01$ *** $p < 0.001$ **** $p < 0.0001$

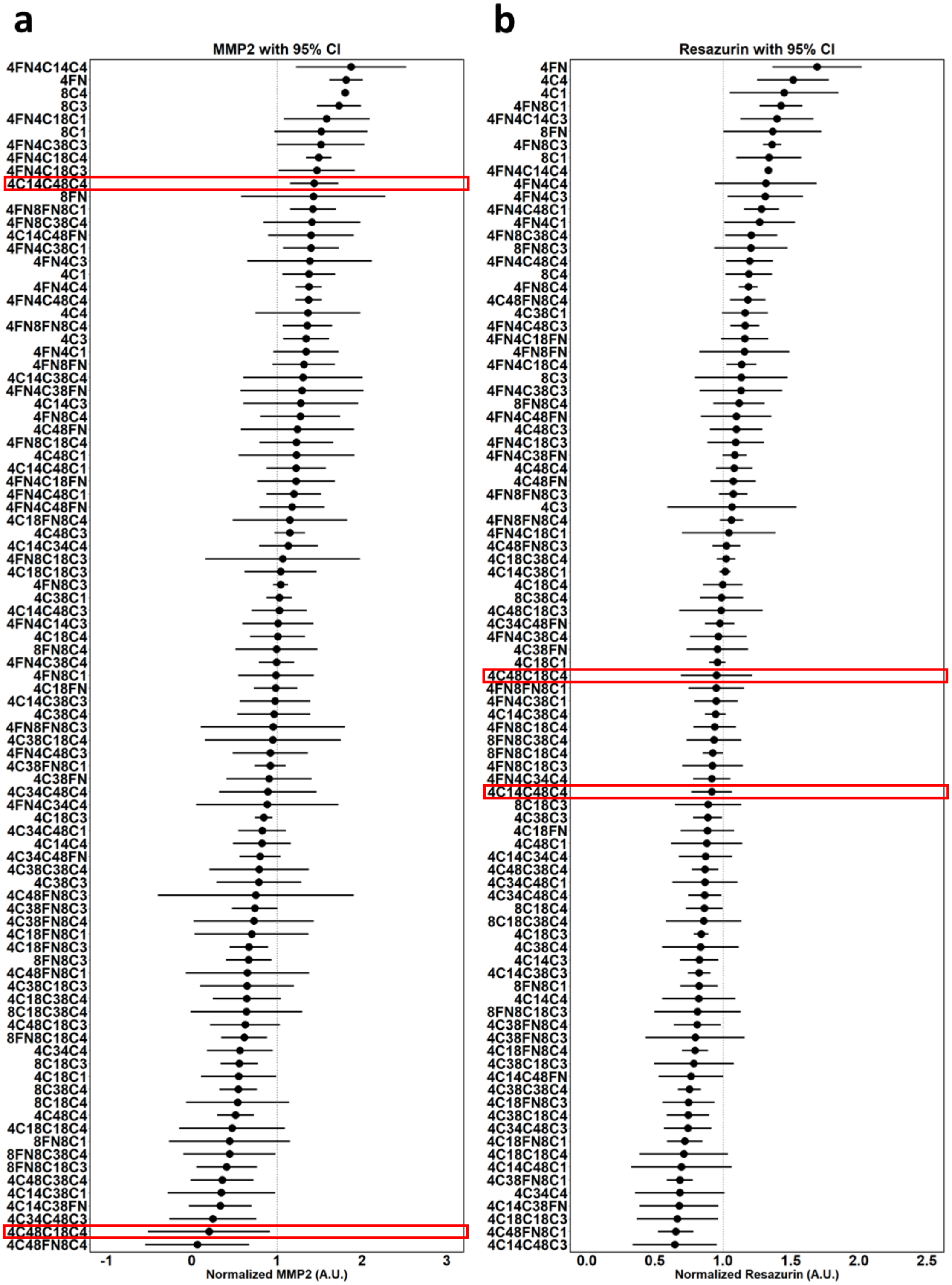

Supplementary Figure 7. Normalized plate reading readouts for all the conditions tested with their corresponding 95% confidence intervals. **a)** MMP2 substrate readout. **b)** Resazurin readout.

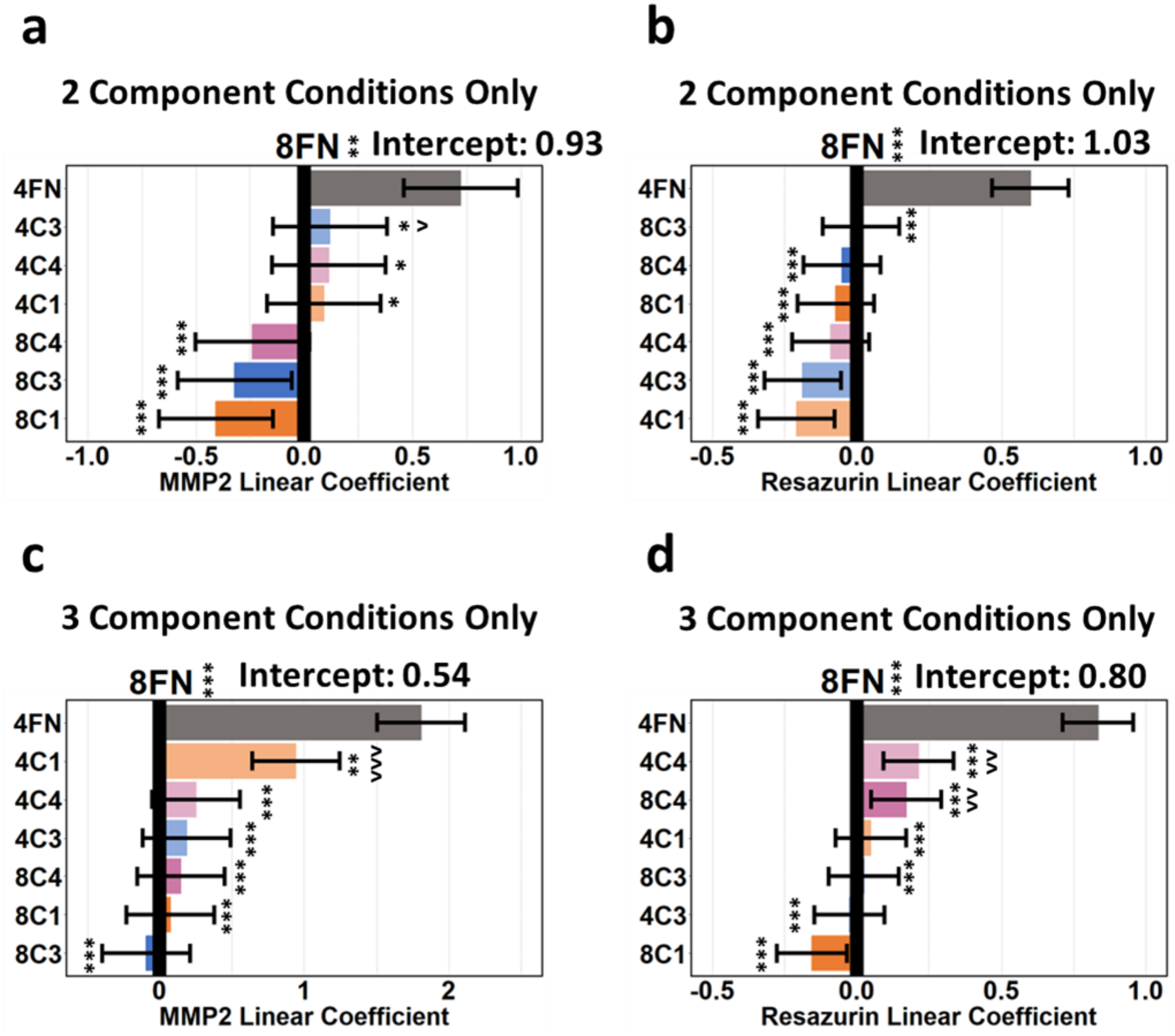

Supplementary Figure 8. Linear regression analysis performed on subsets of plate readout data. **a)** Linear coefficients of MMP2 readout for only 2 component conditions. **b)** Linear coefficients of resazurin readout for only 2 component conditions. **c)** Linear coefficients of MMP2 readout for only 3 component conditions. **d)** Linear coefficients of resazurin readout for only 3 component conditions.
